# A New Approach for Abnormal Human Activities Recognition Based on ConvLSTM Architecture

**DOI:** 10.3390/s22082946

**Published:** 2022-04-12

**Authors:** Roberta Vrskova, Robert Hudec, Patrik Kamencay, Peter Sykora

**Affiliations:** Department of Multimedia and Information-Communication Technologies, University of Zilina, 010 26 Zilina, Slovakia; robert.hudec@uniza.sk (R.H.); peter.sykora@uniza.sk (P.S.)

**Keywords:** dataset, abnormal activities, classification, detection, recognition

## Abstract

Recognizing various abnormal human activities from video is very challenging. This problem is also greatly influenced by the lack of datasets containing various abnormal human activities. The available datasets contain various human activities, but only a few of them contain non-standard human behavior such as theft, harassment, etc. There are datasets such as KTH that focus on abnormal activities such as sudden behavioral changes, as well as on various changes in interpersonal interactions. The UCF-crime dataset contains categories such as fighting, abuse, explosions, robberies, etc. However, this dataset is very time consuming. The events in the videos occur in a few seconds. This may affect the overall results of the neural networks that are used to detect the incident. In this article, we create a dataset that deals with abnormal activities, containing categories such as Begging, Drunkenness, Fight, Harassment, Hijack, Knife Hazard, Normal Videos, Pollution, Property Damage, Robbery, and Terrorism. We use the created dataset for the training and testing of the ConvLSTM (convolutional long short-term memory) neural network, which we designed. However, we also test the created dataset using other architectures. We use ConvLSTM architectures and 3D Resnet50, 3D Resnet101, and 3D Resnet152. With the created dataset and the architecture we designed, we obtained an accuracy of classification of 96.19% and a precision of 96.50%.

## 1. Introduction

Nowadays, video surveillance is widely used in the safety of cities and people. The outputs of the security system have rich information content. This information can be found in the video data. The need for public safety emphasizes the analysis and processing of video data. We focus on increasing security in public areas, and our goal is to simplify the work of the SS (security service). In the direction of abnormal incident classifications, we encounter several problems, namely, insufficient datasets that containing suitable videos for this problem. There are few existing datasets that contain classes such as robbery, abuse, harassment, etc. A good dataset is the foundation of the success of neural networks. Neural learning networks are greatly influenced by datasets that teach them to classify incidents. In order to create an architecture producing the most accurate results, we need a dataset containing information that we can apply to our problem. Therefore, in this article, we not only describe the dataset we created with the name Abnormal Activities Dataset, but also the neural network we proposed.

The authors of [1] deal with the exact understanding and processing of video content at the semantic level. However, interest in the classification of human activities is still growing. In the period marked by COVID-19, the demand for greater protection of our inhabitants is higher. Article [2] also deals with the classification of abnormal human incidents. Its goal is to increase interest in the care of the elderly. The article focuses on the security and remote access needs of our citizens during the COVID-19 pandemic. The authors of [3] are interested in the safety of the elderly. Their goal is to increase security in retirement homes. In [4], the authors are interested in classifying human activities and ensuring human safety. However, even in this article, the authors encountered the problem of low dataset availability, and therefore used a dataset in which people run and walk. Article [5] deals with the issue of classification of abnormal activities without a dataset. The authors try to combine learning with a teacher and learning without a teacher. The monitoring of public space and the automation of this issue are also discussed in [6]. In [7], the authors suggested anomaly detection within video prediction. The authors work with the difference between the predicted future framework and its basic truth. In [8], the authors present AEP (adverse event prediction). AEP derives a prediction model that reveals the correlation between future and present events. The authors also address the issue of recognizing human abnormal incidents in [9], where they deal with the classification of facial expressions. The problem of human activity classification has also been dealt with at the University of Central Florida, where several datasets that contain human activities have been created, such as the UCF11 Action [10] dataset. This dataset contains 11 categories of human activities, such as rocking, walking the dog, etc. Another dataset created at the University of Central Florida is UCF50 [11], where they expanded the UCF11 Action dataset to include basketball and other sports. The dataset was further expanded to create another dataset called UCF101 [12], which contains 101 categories. This dataset is composed of various human activities. However, no dataset contains various violent incidents, and there are no classes such as robbery, battle, kidnapping, etc. Although there is a dataset from this university that deals with such categories—UCF-crime [13]—the dataset is very complicated, because there are a large number of videos in the given classes that do not contain a full-fledged incident, but in which the event only happens for a few seconds of the video, which significantly affected our results. We also know of other datasets that contain various human activities or various abnormal activities in areas or objects where they should not be occurring; these are datasets such as KTH [14], which are still being developed. In [15], authors presented a new dataset, called Human-in-Events or HiEve (human-centric video analysis in complex events). This dataset contains of human motions, poses, and actions in realistic events.

We have been working on this issue for a long time. For example, in previous articles [16,17], we dealt with the classification of human activities. In [16], we applied a 3DCNN (3D convolutional neural network) to datasets such as the UCF YouTube Action dataset, the modified UCF101 dataset, the full UCF50 dataset, and the full UCF101 dataset. In this article, we tested the ConvLSTM architecture on the UCF-crime dataset. In it, we classify video sequences containing different categories of abnormal activities/incidents using our proposed ConvLSTM architecture. We created a dataset for training and testing and called it Abnormal Activities. Section 1 describes the ConvLSTM architecture. Next, we describe the ConvLSTM proposed in the article [18]. We continue by describing the 3D Resnet network, which is also tested on our dataset. We then take a closer look at our proposed neural network architecture. In the Section 2, we describe a dataset that we designed and created called the Abnormal Activities Dataset. In the Section 3, we describe the obtained experimental results. The Section 4 is conclusions and discusion.

## 2. Materials and Methods

Nowadays, there are a number of neural network approaches for video classification. Very often, LSTM (long short-term memory) or 3DCNN are used. In our research, we focus on the ConvLSTM architecture and on creating a dataset that contains abnormal activities.

### 2.1. ConvLSTM Architecture

**Figure 1:**
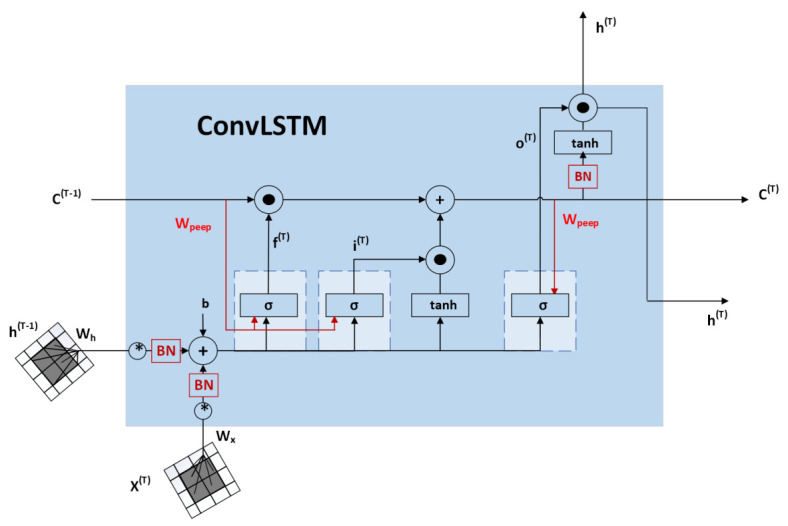
Structure of ConvLSTM [19].

ConvLSTM is a convolutional neural network combined with an LSTM network. Thus, it is similar to LSTM, but convolutional operations are performed at layer transitions. The structure of ConvLSTM can be seen in Figure 1.

ConvLSTM is most suitable for images and videos where time dependence occurs. The network thus captures local spatio-temporal correlations. ConvLSTM determines the future state of a particular cell in the grid using the inputs and past states of its local neighbors. This can be easily achieved by using a convolution operator in state-to-state and input-to-state transitions. The key ConvLSTM [20] equations are given below, where * indicates the convolution operator and ◦ indicates the Hadamard metric:

(1) $$\begin{array}{c} i_{t}=\sigma \left(W_{xi}*X_{t}+W_{hi}*H_{t-1}+W_{ci}C_{t-1}+b_{i}\right), \end{array}$$

(2) $$\begin{array}{c} f_{t}=\sigma \left(W_{xf}*X_{t}+W_{hf}*H_{t-1}+W_{cf}C_{t-1}+b_{f}\right), \end{array}$$

(3) $$\begin{array}{c} C_{t}=f_{t}C_{t-1}+i_{t}tanh\left(W_{xc}*X_{t}+W_{hc}*H_{t-1}+b_{f}\right), \end{array}$$

(4) $$\begin{array}{c} o_{t}=\sigma \left(W_{xo}*X_{t}+W_{ho}*H_{t}+W_{co}C_{t-1}+b_{o}\right), \end{array}$$

(5) $$\begin{array}{c} H_{t}=o_{t}tanh\left(C_{t}\right), \end{array}$$

where $X_{t}$ is input to cell, $C_{t}$ is the cell output, the hidden state of a cell is marked as $H_{t}$, $i_{t},f_{t},o_{t}$ are the input gate, σ is the sigmoidal function, and *W* are convolution kernels [20].

If we look at states as hidden representations of moving objects, ConvLSTM with a larger transition core should be able to capture faster movements, while a network with a smaller core can capture slower movements [20].

### 2.2. ConvLSTM

**Figure 2:**
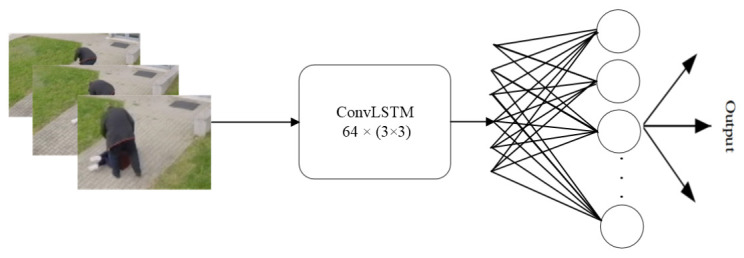
ConvLSTM architecture [18].

As part of the experimental results, we also used the adopted neural network architecture ConvLSTM for testing [18]. This architecture consists of only one ConvLSTM layer, which contains 64 filters and has a kernel size of 3 × 3. Behind the ConvLSTM layer is one Dropout layer with a value of 0.5. This is followed by a Flatten layer and a Dense layer at 256. There is another Dropout layer with a value of 0.5 and the last Dense layer with a number of classes. The architecture of ConvLSTM is shown in Figure 2.

### 2.3. 3D Resnet

**Figure 3:**
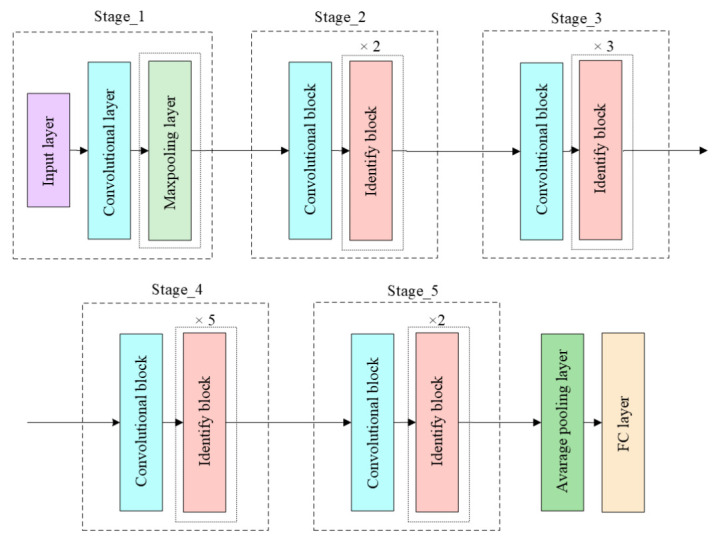
Resnet50 architecture [21].

In general, classic ResNet is used to classify images and uses only 2D convolutional layers on 2D images. However, in this case, 3D convolutional layers are used; these layers are also shifted in the third dimension to extract the related elements between the images. 3D ResNet and the original overall ResNet architecture only replace convolutional 2D layers with convolutional 3D layers; the 2D pooling layer also needs to be replaced with 3D pooling. The classic Resnet50 architecture is shown in Figure 3. In our case, the architectures of 3D Resnet50, 3D Resnet101, and 3D Resnet152 were used. These architectures are different in terms of number of layers [21,22,23].

### 2.4. Proposed Architecture of Neural Network Based on ConvLSTM

The ConvLSTM architecture using ConvLSTM layers has become popular for the extraction of features from video data. For this reason, the ConvLSTM layer in our proposed ConvLSTM was used. The proposed architecture consists of a ConvLSTM layer, a Conv2D layer (convolutional layer for 2D data), and a Time Distributed layer. The ConvLSTM layer contains memory and time continuity. The Conv2D layer is wrapped in the Time Distributed layer.

**Figure 4:**
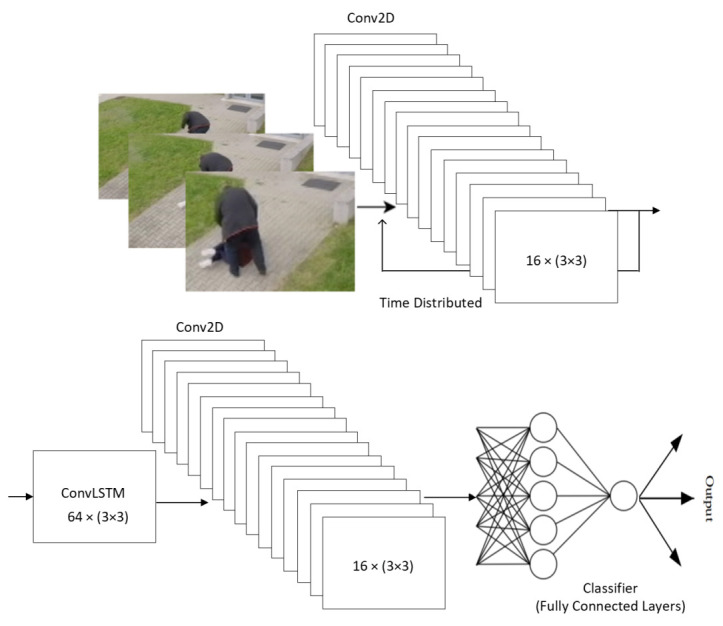
The proposed architecture of ConvLSTM.

The architecture of the proposed ConvLSTM can be seen in Figure 4. This architecture of the proposed ConvLSTM is divided into following layers:

- Convolutional Layer: has a sliding window above data. It is a major building block for convolutional neural network architecture;
- Time Distributed layer: contains memory. This layer attaches the current input to the previous input;
- ConvLSTM layer: contains convolutional and LSTM layers. Function input data are convolutional operation settings;
- Dropout layer: is the regularization part of the neural network. This layer randomly selects neurons that are ignored during training;
- Flatten layer: is the part of the neural network where data are converted from matrix to vector;
- Dense layer: is the last layer, which contains fully connected neurons.

The architecture of the proposed ConvLSTM implemented in this work consists of two Conv2D layers and one ConvLSTM layer. Both Conv2D layers have the same number of filters and kernel size. The input to the first Conv2D layer is images of size 50 × 50 × 3 (height, width, number of channels). The Conv2D layers contain 16 filters with a kernel size of 3 × 3. However. the first Conv2D layer is packaged in the Time Distributed layer. Behind the first Conv2D layer, there is a ConvLSTM layer with a number of filters of 64 and a kernel size of 3 × 3. Finally, there are the Dropout, Flatten, and Dense layers. At the end of the network, there is another pair of Dropout and Dense layers. Both Dropout layers have values of 0.5.

**Figure 5:**
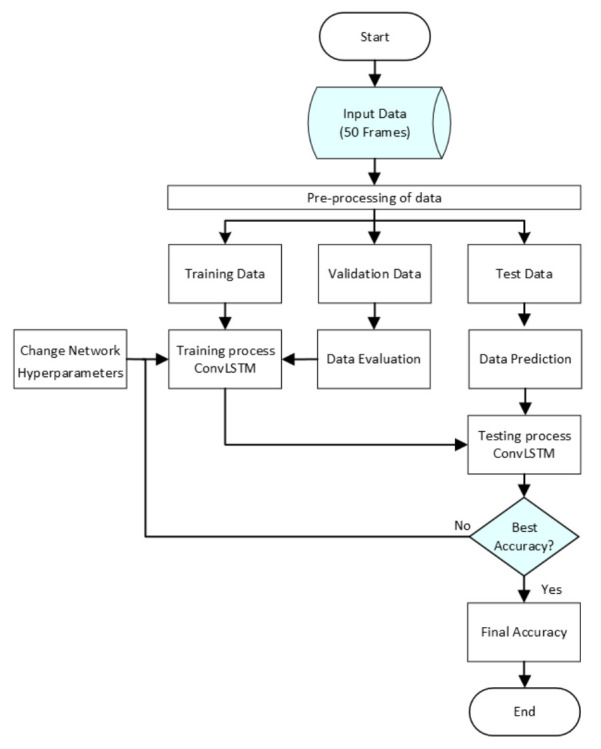
The flowchart of the proposed ConvLSTM.

The flowchart of the proposed ConvLSTM of neural network is shown in Figure 5. The input to the neural network represents the width and height of the image and the number of video frames. Input data were cropped and edited (pre-processing). Data were divided into training, validation, and test data at a ratio of 70:20:10. The training process was run on training data. After that, the testing process was performed on testing data. Finally, the accuracy and confusion matrix were calculated. The output shape of each layer is shown in Table 1 and Table 2. These figures describe output shape, number of total parameters, and number of trainable and non-trainable parameters.

Our proposed ConvLSTM architecture is described using the following pseudo-code (Algorithm 1):
**Algorithm 1** Pseudo-code of the proposed ConvLSTM architecture1:*X* = []2:*Y* = []3:ListClasses = os.listdirinputDir4:*i* = 05:**for***k* in ListClasses **do**6:    Yt=np.zeros(shape=(numClasses))7:    Yt[i]=1;8:    print(k)9:    ListFiles=os.listdir(os.path.join(inputDir,c));10:    **for** *l* in ListFiles **do**11:        frames=framesExtraction(os.path.join(os.path.join(inputDir,c),l))12:    **end for**13:**end for**14:X=np.asarray(X)15:Y=np.asarray(Y)16:X_train,X_test,y_train,y_test=train_test_split(X,Y)17:X_val,x_test,y_val,Y_test=train_test_split(X_test,y_test)18:seq.add(TimeDistributed(Conv2D(filters=16,kernel_size=(3,3)))19:seq.add(ConvLSTM2D(filters=64,kernel_size=(3,3)))20:seq.add(BatchNormalization())21:seq.add(Conv2D(filters=16,kernel_size=(3,3)))22:seq.add(Dropout(0.5))23:seq.add(Flatten())24:seq.add(Dense(256))25:seq.add(Dropout(0.5))26:$seq.compile{(}^{‘}adam^{’},loss={\;}^{‘}categorical\_crossentropy^{’},metrics={[}^{‘}accuracy^{’}])$

Within the neural network, we set the number of epochs to 50 and set the batch size (number of samples that will spread across the network) to 8. The experiments took place on hardware that included a Nvidia graphics card, which is a good choice for deep learning models. This graphics card had advanced hardware support for specific deep learning calculations. All of the experimental results were performed on an NVIDIA GeForce GTX 1660 Ti graphics card. Neural network architecture was programmed in the Python language with frameworks such as Keras and TensorFlow.

## 3. Experimental Results

In this section, the obtained experimental results are described. A new dataset for classification of non-standard human behavior was created. This new dataset was created because there is no other appropriate dataset for recognition and classification of non-standard human behavior. The obtained experimental results using the proposed architecture of ConvLSTM were compared with results from four well-known neural networks (ConvLSTM, 3D Resnet50, 3D Resnet101 and 3D Resnet152).

### 3.1. Abnormal Activities Dataset

**Figure 6:**
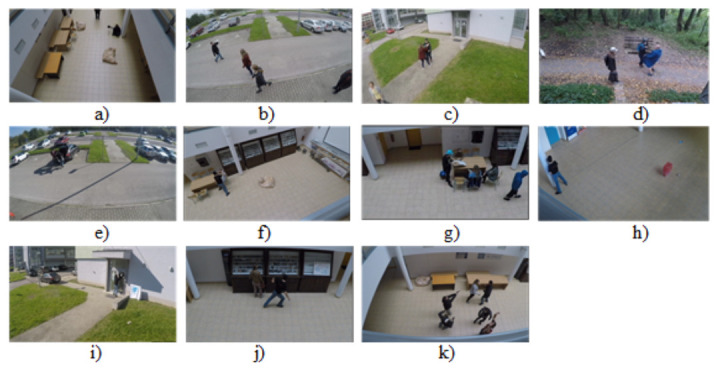
Abnormal Activities Dataset: (**a**) Begging, (**b**) Drunkenness, (**c**) Fight, (**d**) Harassment, (**e**) Hijack, (**f**) Knife Hazard, (**g**) Normal Videos, (**h**) Pollution, (**i**) Property Damage, (**j**) Robbery, (**k**) Terrorism.

As part of our abnormal behavior classification research, we created a dataset called the Abnormal Activities Dataset. The dataset consists of videos with ten non-professional actors. The dataset contains 1069 videos. This dataset was divided into eleven classes (Begging, Drunkenness, Fight, Harassment, Hijack, Knife Hazard, Normal Videos, Pollution, Property Damage, Robbery, Terrorism), as shown in the Figure 6. These scenes were performed in different lighting conditions. During the creation of the dataset, our goal was to simulate the layout of the security camera system in public places such as in buildings, in parking lots, and in nature. The Abnormal Activities Dataset provides diversity mainly in terms of actions in real environments with the presence of different variations in camera motion, appearance and position of the examined object, different illumination conditions, etc. The complexity of the dataset is influenced by the choice of categories, some of which are very similar, such as the Knife Hazard and Fight categories. In the mentioned categories, the classification can be influenced by similar reactions of people to fighting, but also to being threatened with a knife, as well as similar movements during attacking and fighting. The categories of Drunkenness and Begging can also be very similar, as the drunkard also appears to annoy the people around him. These similarities between categories make the dataset we created more difficult to classify. Furthermore, all the categories created contain entries from different angles and backgrounds. Each category contains about 100 videos. In each video in all categories, the event takes place for the entire video, so the length of the videos ranges from about 1 s to 30 s. Videos in the same category may have some features in common, such as the same person, similar or the same background, and similar or the same camera angle. Uploaded videos are in .avi format. All videos were recorded by two different cameras. The first camera was the GoPro Hero 4 and the second was the Lamax X7. The GoPro Hero4 camera can record in 4 K resolution (UltraHD). It also supports lower video resolutions such as 1080 p, 720 p, and 480 p. It can record up to 120 frames per second, which is excellent for recording fast movements. The Lamax 7 camera can record in native 2.7 K resolution. It also supports lower video resolutions such as 1080 p, 720 p, and 480 p. It can record 60 frames per second.

The Abnormal Activities Dataset consists of the following categories: Begging, Drunkenness, Fight, Harassment, Hijack, Knife Hazard, Normal Videos, Pollution, Property Damage, Robbery, Terrorism. The aforementioned categories can be seen in Figure 6.

Specification and description of individual categories:

- Knife Hazard: The video contains scenes where a person threatens his surroundings with a knife;
- Robbery: We can see different types of robbery in the videos;
- Pollution: The videos contain people throwing trash, polluting their surroundings;
- Fight: The category contains videos where a fight takes place between two or more people;
- Harassment: The videos contain scenes with inappropriate gestures, as well as direct unwanted contact with people and exposure to the public;
- Property damage: The videos contain people who are damaging someone else’s property;
- Hijack: The videos show various abductions of one person from a crowd, as well as a lone person;
- Terrorism: The videos show actions where a person places a bag with a bomb among people or shows a bomb on himself, or where the person starts shooting at people around him;
- Begging: The videos feature people who annoy or harass surrounding people by begging or asking for money;
- Normal videos: These are videos where no abnormal activity occurs;
- Drunkenness: There are people in the videos who obviously have problems with balance, stability, and drunkenness.

The already designed architectures were subsequently tested on the designed Abnormal Activities Dataset.

### 3.2. AIRTLab Dataset

**Figure 7:**
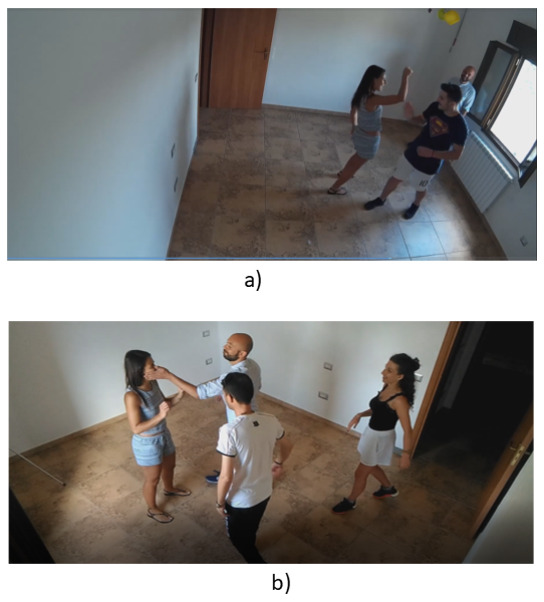
AIRTLab Dataset: (**a**) nonviolent, (**b**) violent.

The AIRTLab dataset was developed to specifically test the robustness of violence detection techniques against false positives in nonviolent fast-moving clips (such as hugs, clapping, teasing, etc.). The dataset is publicly available. The dataset consists of 350 clips that are in MP4 format (H.264 codec) with an average length of 5.63 s, with the shortest video lasting 2 s and the longest 14 s. For all clips, the resolution is 1920 × 1080 pixels and the frame rate is 30 fps. The dataset is divided into two main directories, “nonviolent” and “violent”, which include clips marked as showing nonviolent behavior and violent behavior, respectively, as can be seen in Figure 7.

- Nonviolent: contains 120 clips representing nonviolent behavior, from two angles;
- Violent: contains 230 clips representing violent behavior, from two angles.

All clips were recorded in the same room, with natural lighting conditions, by placing two cameras in two different places (upper left corner in front of the room’s door and upper right corner on the side of the door). The clips were played by a group of non-professional actors ranging from two to four people per clip. In the violent clips, the actors were asked to simulate actions common in battles, such as kicks, punches, slaps, batting, stabbing, and pistol shots. In the case of nonviolent clips, actors were asked to simulate actions that could lead to false-positive results in violence detection techniques due to speed of movement or similarity to violent actions. Specifically, nonviolent clips include actions such as hugging, clapping, cheering, and gesturing. All actions in both violent and nonviolent clips were manually annotated. The dataset was adapted to specifically test resistance to false-positive results [18].

### 3.3. Results

**Figure 8:**
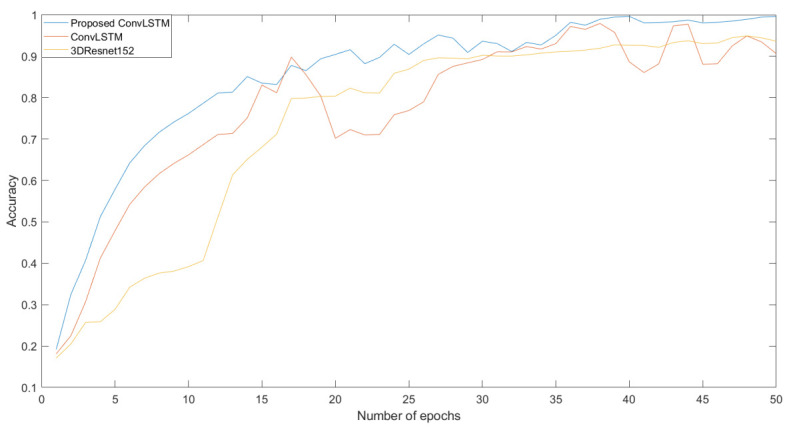
Comparison of accuracy during training process (ConvLSTM, 3D Resnet152, and proposed ConvLSTM).

A dataset (Abnormal Activities Dataset) was used to test the neural network architectures mentioned in the previous chapter. The dataset was divided into three sets—training, testing, and validation—at a ratio of 70:20:10. The training set contained 748 videos, which represent 70% of the data. The testing set contained 214 videos, which represent 20% of the dataset. The validation set contained 107 videos, which represent 10% of the data. The neural network model learned to classify videos into 11 classes. We used the already mentioned architectures for classification. We compared the result with our dataset and the neural network architecture we created with other already proposed architectures such as 3D Resnet50, 3D Resnet101, and 3D Resnet152, as well as ConvLSTM. We only used 3D Resnet architectures, with no pre-trained model. The length of the sequence was 50 frames and the size of the image was 50 × 50 × 3 (the final number indicates that the image has three channels) at the input of the networks. Videos were pre-processed to these dimensions. The comparison of the training process between the proposed neural networks, ConvLSTM, and 3D Resnet152 can be seen in Figure 8.

As we can see, each neural network’s training started at a different loss value. However, we are interested in which loss function had the largest decrease. We can see that the decline was rapidly large. Accuracy grew from 0 to 1 in all cases. However, in some cases, it was smoother and more even, while elsewhere, there were decreases and sudden increases in the given epoch. After the testing process, we processed the resulting metrics for each neural network architecture into a confusion matrix. The confusion matrix tells us how a video was classified into a given category. Subsequently, we analyzed each confusion matrix separately for each neural network architecture.

Continuing, we generated a confusion matrix of the first neural network architecture taken by ConvLSTM [18,21,22,23]. This confusion matrix can be seen in Table 3. The videos are classified into the following categories: 1—Begging, 2—Drunkenness, 3—Fight, 4—Harassment, 5—Hijack, 6—Knife Hazard, 7—Normal Videos, 8—Pollution, 9—Property Damage, 10—Robbery, 11—Terrorism. We can observe that the architecture of the neural network had the greatest problem with category number 4, Harassment. Here, it correctly classified only two videos and categorized the others as 8, Pollution, and 10, Robbery. The Harassment class has many similar features with Robbery. A person most often looks for one person, most often they are alone, and the reaction is quick and unpleasant. While Pollution can be very similar, it also contains people who are trying to be inconspicuous.

We also applied a 3D Resnet network. We tested 3D Resnet50, 100, and 152. For each of these networks, we generated a confusion matrix so that we could observe how the classification accuracy increased with increasing number of layers. The first 3D Resnet50 network had a classification problem; the overall accuracy was only 36.19%. Thus, we generated a confusion matrix, which can be seen in Table 4.

As we can see the confusion matrix is scattered. The neural network most often classified videos into categories 3. Fight and 9. Property damage. Clear and distinctive features are in these categories. The neural network could classify obscure features into these categories because it could not predict what the other might be. We also tested 3D Resnet101, where we can already see a significant increase in accuracy but also straightness as the number of layers increases with increasing accuracy. In this case we have achieved accuracy 61.91%. The generated confusion matrix can be seen in the Table 5.

We see fewer classification errors in this confusion matrix. Again, the largest problem with classification was with class 4, Harassment. The harassment class may contain features that resemble multiple features in other classes. For example, harassment may resemble robbery, and hijacking, but also begging. The rising trend of accuracy led us to test the 3D Resnet152. As we expected, the accuracy in this case increased to 90.47%. Again, we can observe a directly proportional increase in accuracy with increasing layers. At the same time, we can evaluate that with every 50 layers, our accuracy increased by 30%. The confusion matrix after testing 3D Resnet 152 can be seen in the Table 6.

The confusion matrix after testing 3D Resnet152 showed promising results. We see that the classification errors occurred in class 4, Harassment, with, however, a smaller number of errors than in the previous case. An error also occurred during classification into category 7, Normal Videos. There were minimal classification errors in the other categories. Subsequently, we tested our designed architecture on the dataset we created. We also generated the resulting confusion matrix. The overall results were the best compared to other architectures. In this case, we achieved a classification accuracy of 96.19%. The results of the confusion matrix can be seen in Table 7.

As we can see, the classification error was minimal. During the classification, this time the neural network did not fail once in the classification of the Harassment class. The neural network was mistaken only once in class 7, Normal Videos, and once in class 8, Pollution. It made a mistake twice in class 11, Terrorism.

For all results of the experiments, we also evaluated the resulting evaluations such as F1 score, precision, and recall. We then compared the evaluation criterion in Table 8. We compared the adopted architectures with the architecture we designed. All experiments were performed under the same conditions, with the same batch size and number of epochs. We inserted the same size pictures and the same number of frames for the input.

The proposed architecture of ConvLSTM achieved the best results (evaluation metrics) on the created dataset. To better represent our results, we created Table 9, where we can see the resulting accuracy and precision values for all the architectures used on our dataset.

We achieved the best value for all evaluation metrics, which proves that the dataset we created is usable within the range of abnormal behaviors of people and. at the same time, that we have designed an architecture that can accurately classify these categories in the dataset to an accuracy of 96.19%. In the results of 3D Resnet networks, we can observe a linear increase in accuracy. Each increase in the number of layers increases the average accuracy by 30%. In this case, we can evaluate that 3D networks are usable for video classification, but with such a demanding dataset, they achieve worse results than networks containing ConvLSTM layers.

We also tested the proposed architecture and the three networks with the best results—ConvLSTM, 3D Resnet152, and our proposed architecture, ConvLSTM—on the freely available AIRTLab dataset. This dataset is mainly used to test architectures to see if violent or a nonviolent incident in a video is correctly classified. We compared the obtained results in terms of the evaluation metrics of accuracy and F1 score. We also generated confusion matrices to compare the results, which can be seen in Table 10, Table 11 and Table 12.

In the case of the first confusion matrix, we observe that during the classification, an error occurred in which three nonviolent videos were classified into the violent class. In the second case, three videos in the violent class were incorrectly classified as nonviolent.

The best results were obtained with the ConvLSTM architecture, which correctly ranked all videos in the nonviolent class. In the violent class, only two videos were classified as nonviolent.

The proposed architecture in this case classified two violent videos into the nonviolent class and two nonviolent videos into the violent class. The resulting values of the evaluation metrics accuracy and F1 score can be seen in Table 13.

The best results on the AIRTLab dataset were achieved by the proposed ConvLSTM architecture. The proposed ConvLSTM achieved an accuracy of 98.29% and a F1 score of 97.66%. When comparing the results with the previous dataset, we can observe a significant change in evaluation metrics for the ConvLSTM network and the proposed ConvLSTM architecture. The difference in results for the ConvLSTM architecture could be due to large differences in the datasets. On the AIRTLab dataset, the tested architectures obtained significantly better results because the dataset contained only two classes with 350 videos. The dataset we created was therefore more demanding for the ConvLSTM architecture. The complexity of the Abnormal Activities Dataset was also confirmed by the 3D Resnet152 architecture, which achieved an accuracy of 90.47% on our dataset and an accuracy of 91.42% on the AIRTLab dataset.

## 4. Conclusions and Discussion

In this paper, we created dataset called the Abnormal Activities Dataset. We proposed a neural network architecture for classifying human activities from video using ConvLSTM architecture. We used our created dataset for training and testing. Classification was carried out for 11 classes: Begging, Drunkenness, Fight, Harassment, Hijack, Knife Hazard, Normal Videos, Pollution, Property Damage, Robbery, and Terrorism. Videos were cropped to an input size of 50 × 50 × 3 RGB. We also tested other architectures on our designed dataset. We tested ConvLSTM from [18], as well as 3D Resnet50, 3D Resnet101, and 3D Resnet152. The mentioned 3D Resnet architectures were not pre-trained. We only used the architectures to train new model. We compared the results of all architectures on the dataset we created. We compared the evaluation metrics of accuracy, and precision. We also compared interim results during testing. We processed these results into graphs, which can be seen in Figure 8. To compare the results, we also generated a confusion matrix for each neural network architecture. In all cases, we evaluated that the neural network we designed achieved the best results on the dataset we created.

For the overall results using the Abnormal Activities Dataset, the evaluation metrics (accuracy, precision, recall, and F1 score) were obtained. The overall accuracy was 96.16%. Precision reached a value of 96.50% and recall reached 96.85%. The F1 score was 96.42%. The results clearly confirm that the proposed neural network architecture is suited for the classification of human activities.

For a more complex comparison of the proposed neural network (ConvLSTM), we tested our dataset on another architectures. However, other architectures did not perform as well as the architecture we proposed. The best results from other architectures were achieved by the neural network architecture ConvLSTM. ConvLSTM, proposed by other authors, achieved an accuracy of 92.38% and a precision of 89.68% on our dataset. 3D Resnet152 also approached these results. The 3D Resnet152 architecture achieved an accuracy of 90.47% and a precision of 91.36%.

The main contribution of this article is in the field of recognition and classification of abnormal human activities in public and private spaces. This real-time monitoring can be used in various applications. For our proposed architecture, the overall accuracy of the proposed neural network (96.16% for our generated data set) is sufficient. It was also necessary to create the aforementioned dataset, due to the lack of datasets on this issue. The use of this proposed solution (proposed architecture ConvLSTM) is applicable in the field of security, medicine, and in the monitoring and classifying of non-standard behavior of people in public places such as parks, railway stations, and city squares. The result of this work will aid warning systema for security staff in the event of non-standard behavior of people in these areas of interest.

## Figures and Tables

**Table 1:** The description of the layers of the proposed ConvLSTM.

Layer (Type)	Output Shape [%]	Parameters
Time Distributed (Conv2D layer)	(None, 50, 50, 50, 16)	448
ConvLSTM layer	(None, 50, 50, 50, 64)	184,576
Batch normalization	(None, 50, 50, 50, 64)	256
Conv2D layer	(None, 50, 50, 50, 16)	9232
Dropout	(None, 50, 50, 50, 16)	0
Flatten	(None, 2,000,000)	0
Dense	(None, 256)	512,000,256
Dropout	(None, 256)	0
Dense	(None, 11)	2827

**Table 2:** The values of the parameters of the proposed ConvLSTM.

Total parameters	512,197,595
Trainable parameters	512,197,467
Non-Trainable parameters	128

**Table 3:** The confusion matrix for ConvLSTM [18].

Targeted/Predicted	1	2	3	4	5	6	7	8	9	10	11
1	14	0	0	0	0	0	0	0	0	0	0
2	0	7	0	0	0	1	0	0	0	0	0
3	0	0	13	0	0	0	0	0	0	0	0
4	0	0	0	2	0	0	0	2	0	1	0
5	0	0	0	0	8	0	0	0	0	0	0
6	0	0	0	0	0	12	0	0	0	0	0
7	0	0	0	1	0	0	8	0	0	0	0
8	0	0	0	0	0	0	1	12	0	0	0
9	0	0	0	0	0	0	0	0	6	0	0
10	0	0	0	1	0	0	1	0	0	7	0
11	0	0	0	0	0	0	0	0	0	0	8

**Table 4:** The confusion matrix for 3D Resnet50 [21].

Targeted/Predicted	1	2	3	4	5	6	7	8	9	10	11
1	5	0	5	0	0	0	0	0	0	0	0
2	0	0	0	0	0	0	0	0	8	0	0
3	0	0	15	0	0	0	0	0	0	0	0
4	0	0	0	0	0	0	0	0	7	0	0
5	0	0	1	0	3	0	0	0	3	0	0
6	0	0	3	0	0	0	0	0	5	0	0
7	0	0	4	0	0	0	1	0	7	2	0
8	0	0	6	0	0	0	0	0	3	0	0
9	0	0	2	0	0	0	0	0	9	0	0
10	0	0	1	0	0	0	1	0	3	3	0
11	0	0	0	0	0	0	0	0	7	0	2

**Table 5:** The confusion matrix 3D Resnet101 [21].

Targeted/Predicted	1	2	3	4	5	6	7	8	9	10	11
1	9	0	0	0	0	0	0	0	0	0	0
2	1	2	3	0	0	3	0	0	0	0	0
3	2	0	7	0	0	0	0	0	0	0	0
4	0	0	2	1	0	2	0	0	0	0	0
5	0	0	0	0	6	4	0	0	0	0	0
6	0	0	0	0	0	11	0	0	0	0	0
7	2	0	0	0	0	0	11	0	0	0	0
8	1	0	1	0	1	4	2	8	0	0	0
9	3	0	2	0	0	0	0	0	2	0	1
10	1	0	0	0	0	1	0	0	0	2	2
11	0	0	0	0	0	2	0	0	0	0	6

**Table 6:** The confusion matrix for 3D Resnet152 [21].

Targeted/Predicted	1	2	3	4	5	6	7	8	9	10	11
1	8	0	0	0	0	0	0	0	0	0	0
2	0	5	0	0	0	0	0	0	0	0	0
3	1	0	9	0	0	0	0	0	0	0	0
4	0	0	0	6	0	0	0	2	0	2	0
5	0	0	0	0	10	0	0	0	0	0	0
6	0	0	0	0	0	8	0	0	0	0	0
7	0	0	0	0	0	0	9	4	0	0	0
8	0	0	0	0	0	0	0	14	0	0	0
9	0	0	0	0	0	0	0	0	12	0	0
10	0	0	0	0	0	0	0	0	0	8	0
11	0	0	0	0	1	0	0	0	0	0	6

**Table 7:** The confusion matrix for ConvLSTM architecture.

Targeted/Predicted	1	2	3	4	5	6	7	8	9	10	11
1	12	0	0	0	0	0	0	0	0	0	0
2	0	6	0	0	0	0	0	0	0	0	0
3	0	0	9	0	0	0	0	0	0	0	0
4	0	0	0	5	0	0	0	0	0	0	0
5	0	0	0	0	15	0	0	0	0	0	0
6	0	0	0	0	0	8	0	0	0	0	0
7	0	0	0	1	0	0	15	0	0	0	0
8	0	0	0	0	0	0	1	9	0	0	0
9	0	0	0	0	0	0	0	0	8	0	0
10	0	0	0	0	0	0	0	0	0	7	0
11	0	0	0	0	2	0	0	0	0	0	7

**Table 8:** The evaluation criterion of the architectures using our dataset.

Evaluation Metrics	ConvLSTM	3D Resnet50	3D Resnet101	3D Resnet152	Proposed ConvLSTM
Precision [%]	89.68	31.54	58.64	91.36	96.50
Recall [%]	90.50	47.07	79.25	93.62	96.85
F1 score [%]	89.93	28.42	57.27	91.31	96.42

**Table 9:** Comparison of accuracy obtained by different neural network architectures using our dataset.

Architectures for Video Recognition	Accuracy [%]	Precision [%]
ConvLSTM [18]	92.38	89.68
3D Resnet50 [21]	36.19	31.54
3D Resnet101 [21]	61.91	58.64
3D Resnet152 [21]	90.47	91.36
Proposed architecture of ConvLSTM	96.19	96.50

**Table 10:** The confusion matrix for 3D Resnet152 architecture on the AIRTLab dataset.

Targeted/Predicted	1	2
1	21	3
2	3	43

**Table 11:** The confusion matrix for ConvLSTM architecture on the AIRTLab dataset [18].

Targeted/Predicted	1	2
1	22	2
2	2	44

**Table 12:** The confusion matrix for our proposed ConvLSTM architecture on the AIRTLab dataset.

Targeted/Predicted	1	2
1	23	1
2	1	45

**Table 13:** Comparison of accuracy obtained by different neural network architectures using the AIRTLab dataset [18].

Architectures for Video Recognition	Accuracy [%]	F1 Score [%]
ConvLSTM [18]	97.15	96.89
3D Resnet152 [21]	91.42	90.49
Proposed architecture of ConvLSTM	98.29	97.66

## Data Availability

The data presented in this study are available on request from the corresponding author. This is according to laboratory rules.

## Abbreviations

The following abbreviations are used in this manuscript:

LSTM	Long Short-Term Memory
ConvLSTM	Convolutional Long Short-Term Memory
Conv2D	Convolutional 2D layer
3DCNN	3D Convolutional Neural Network
UCF	University of Central Florida
